# Cone-beam computed tomography assessment of the root canal morphology of primary molars

**DOI:** 10.1186/s12903-023-03414-z

**Published:** 2023-09-25

**Authors:** Afsaneh Rahmati, Elham Khoshbin, Abbas Shokri, Hadis Yalfani

**Affiliations:** 1 Endodontic department, School of Dentistry, Hamadan University of Medical Sciences, Hamadan, Iran; 2 Department of Oral and Maxillofacial Radiology, Dental Implants Research Center, Faculty of Dentistry, Hamadan University of Medical Sciences, Hamadan, Iran; 3 Private Practice, Hamadan, Iran

**Keywords:** Tooth root, Deciduous, Cone-beam computed tomography

## Abstract

**Background:**

This study aimed to assess the root canal morphology of primary molars using cone-beam computed tomography (CBCT).

**Methods:**

This cross-sectional study evaluated 60 maxillary and mandibular primary first and second molars on CBCT scans of patients retrieved from the archives of Hamadan School of Dentistry between 2018–2020. The teeth were evaluated regarding the number of roots and canals, canal type according to the Vertucci’s classification, and root surface concavities. Data were analyzed descriptively and by independent t-test.

**Results:**

The most frequent number of canals and roots in the maxillary right and left first molars was 3 canals (60%) and 3 roots (80%). These values were 4 canals (80%) and 5 canals (50%) with 3 roots in the maxillary right and left second molars, respectively, 4 canals (100%) and 2 roots (50%), and 3 canals (60%) and 2 roots (50%) in mandibular right and left first molars, respectively, and 4 canals (92.3%) and 3 roots (61.5%) in mandibular right and left second molars. Vertucci’s type IV was the most common canal type in mesial and distal canals, type I was the most common in mesiobuccal, mesiolingual, distobuccal, and distolingual, and types I and II were the most common in the palatal canal. The maximum and minimum concavities were noted in the buccal (26.7%) and mesial (8.3%) surfaces, respectively.

**Conclusions:**

A wide variation exists in the number of roots and canals of maxillary and mandibular primary molars, which calls for further attention in treatment of such teeth.

## Background

Early loss of primary teeth, particularly primary molars, can cause several complications such as narrowing of dental arch, over-eruption of opposing teeth, and impaired occlusion [1]. Endodontic treatment can help preserve such teeth. However, a successful endodontic treatment requires complete cleaning and shaping of all parts of the root canal system along with a coronal restoration with hermetic seal [1]. However, this cannot be easily achieved in oval or ribbon-shaped root canals [2].

Poor knowledge about the internal root canal anatomy is among the most important factors responsible for endodontic treatment failure [1, 3]. Variations exist in root canal anatomy and morphology among different populations as well. Such variations depend on several factors such as ethnicity, race, genetics, age, and gender [4]. Thus, comprehensive knowledge about the root canal anatomy and its variations in different teeth and also in different populations is imperative for a successful endodontic treatment [1, 3, 5, 6]. Root canal anatomy does not normally follow a uniform conical shape, and there are often additional canals, anastomosis, and irregularities that need to be taken into account. Also, variations in the number and type of canals are among the most common root canal abnormalities [5].

Pulpectomy in primary teeth is performed aiming to ensure normal physiological exfoliation of the respective tooth and subsequent eruption of its permanent successor. Also, pulpectomy is performed to guarantee long-term service of a tooth with pulpal involvement, which has yet to be normally exfoliated. However, comprehensive knowledge about the root and canal morphology of primary teeth is imperative for this procedure since primary teeth have a wide range of unpredictable anatomical variations [7–11].

The majority of complications encountered in treatment of primary teeth are related to their unique morphology, and having a comprehensive knowledge about their anatomical complexities and variations can help minimize such complications [12, 13].

Cone-beam computed tomography (CBCT) is an advanced imaging modality than can provide highly accurate 3D information [14]. Considering all the above, this study aimed to assess the root canal morphology and characteristics of primary molars like the number of root canals, number of roots, distribution of different root canal types and root surface concavities, and Comparison of the variables between the first and second primary molars using CBCT.

## Methods

This cross-sectional study was conducted on eligible CBCT scans of patients available in the archives of School of Dentistry, Hamadan University of Medical Sciences from 2018 to 2020. The CBCT scans had been taken for diagnostic purposes not related to this study. The study protocol was approved by the ethics committee of the university (IR.UMSHA.REC.1399.856).

The sample size was calculated to be 59 according to the Krejcie and Morgan’s table for sample size calculation.

The inclusion criteria were available CBCT scans of children younger than 8 years of age, presence of all maxillary and mandibular first and second molars in dental arch, intact maxillary and mandibular first and second molars with no pathology or periapical lesion, and no history of endodontic treatment in maxillary and mandibular first and second molars.

The exclusion criteria were poor-quality CBCT scans, and teeth with external root resorption. A total of 60 CBCT scans for each tooth were selected by convenience sampling. All CBCT images had been obtained by New Tom 3G CBCT scanner with the exposure settings of 9–14 mA, 110 kVp, 0.2–0.4 mm voxel size, exposure time of 6 s, and 17 × 20 or 10 × 20 cm field of view. The images were inspected in axial, coronal and sagittal sections with 3 mm slice thickness and 3 mm slice interval by a dental student and an oral and maxillofacial radiologist with 10 years of clinical experience using New Tom NNT Viewer (Verona, Italy) software. The images were observed on a 20-inch monitor (LG, Seoul, Korea) in a completely dark room and the observers were allowed to change the contrast and brightness settings to optimize the viewing conditions as desired. Initially, the observers were trained independently on how to use the software to examine the images and evaluate the morphology of the roots, and they were calibrated for image assessment. Two observers examined all samples’ scans twice at two-week intervals. Agreement between the two observers was assessed.

The intra-class correlation coefficient (ICC) was used to control inter-observer and intra-observer agreements. The significance level was set at 0.05.

The intra-observer and interobserver agreements were above 90%, indicating excellent agreement.

The number of roots, number of canals, and root surface concavities were separately evaluated on axial and sagittal sections for each of the primary maxillary and mandibular right and left first and second molars. Canal type was also determined on axial sections according to the Vertucci’s classification [15].

The data were analyzed using SPSS version 23. The data were tabulated, frequency, percentage and mean and standard deviation values were reported, and statistical analysis was performed using independent t-test.

## Results

Table 1 presents the frequency distribution of the number of root canals in primary molars. As shown, four canals had the highest frequency in maxillary right second molars (80%), mandibular left second molars (100%), mandibular left first molars (66.7%), mandibular right first molars (100%), and mandibular right second molars (92.3%). Five canals had the highest frequency in maxillary left second molars and maxillary right second molars. The majority of maxillary left first molars (60%) had three canals.

**Table 1:** Frequency distribution of the number of root canals in primary molars

Frequency of canals	Maxillary right second molar Number (%)	Maxillary right first molar Number (%)	Maxillary left first molar Number (%)	Maxillary left second molar Number (%)	Mandibular left second molar Number (%)	Mandibular left first molar Number (%)	Mandibular right first molar Number (%)	Mandibular right second molar Number (%)
3 canals	(10) 1	(60) 3	(60) 3	(25) 2	(0) 0	(33.3) 2	(0) 0	(0) 0
4 canals	(80) 8	(40) 2	(20) 1	(25) 2	(100) 9	(66.7) 4	(100) 4	(92.3) 12
5 canals	(10) 1	(0) 0	(20) 1	(50) 4	(0)	(0) 0	(0) 0	(7.7) 1
Total	(100) 100	(100) 5	(100) 5	(100) 8	(100) 9	(10) 6	(100) 4	(100) 13

Table 2 indicates the frequency distribution of the number of roots in primary molars. Three roots had the highest frequency in maxillary right second molars (80%), maxillary right first molars (80%), maxillary left first molars (80%), maxillary left second molars (100%), mandibular left second molars (66.7%), mandibular left first molars (50%), and mandibular right second molars (61.5%). Also, 50% of mandibular left first molars and mandibular right first molars had 2 roots. Four roots had the highest frequency in maxillary right second molars (20%), mandibular left second molars (22.2%), mandibular right first molars (25%), and mandibular right second molars (30.8%).

**Table 2:** Frequency distribution of the number of roots in primary molars

Roots	Maxillary right second molar Number (%)	Maxillary right first molar Number (%)	Maxillary left first molar Number (%)	Maxillary left second molar Number (%)	Mandibular left second molar Number (%)	Mandibular left first molar Number (%)	Mandibular right first molar Number (%)	Mandibular right second molar Number (%)
2 canals	(0) 0	(20) 1	(20) 1	(0) 0	(11.1) 1	(50) 3	(50) 2	(7.7) 1
3 canals	(80) 8	(80) 4	(80) 4	(100) 8	(66.7) 6	(50) 3	(25) 1	(61.5) 8
4 canals	(20) 2	(0) 0	(0) 0	(0) 0	(22.2) 2	(0) 0	(25) 1	(30.8) 4
Total	(100) 10	(100) 5	(100) 5	(100) 8	(100) 9	(10) 6	(100) 4	(100) 13

Table 3 presents the frequency distribution of different root canal types in primary molars. The majority of primary teeth did not have the mesial (85%) canal. This rate was 76.7% for the distal canal, 68.3% for the mesiolingual canal, 73.3% for the distolingual canal, and 55% for the palatal canal.

**Table 3:** Frequency distribution of different root canal types in primary molars

Canal	Mesial Number (%)	Distal Number (%)	Mesiobuccal Number (%)	Mesiolingual Number (%)	Distobuccal Number (%)	Distolingual Number (%)	Palatal Number (%)
type							
Not having this canal	(85) 51	(76.7) 46	(15) 9	(68.3) 41	(25) 15	(73.3) 44	(55) 33
Type I	(3.3) 2	(1.7) 1	(70) 42	(31.7) 19	(68.3) 38	(20) 12	(18.3) 11
Type II	(0) 0	(3.3) 2	(5) 3	(0) 0	(3.3) 2	(5) 3	(18.3) 11
Type III	(0) 0	(18.3) 11	(3.3) 2	(0) 0	(0) 0	(0) 0	(0) 0
Type IV	(11.7) 7	(0) 0	(6.7) 4	(0) 0	(8.3) 5	(1.7) 1	(8.3) 5
Total	(100) 66	(100) 60	(100) 60	(100) 60	(100) 60	(100) 60	(100) 60

Vertucci’s type I was the most common canal type in mesiobuccal (70%) and distobuccal (68.3%) root canals. Type II had the highest frequency in the palatal (18.3%) and mesiobuccal (5%) root canals. Type III was the most common type in distal (18.3%) followed by mesiobuccal root canals (3.3%). Type IV had the highest frequency in mesial (11.7%) followed by distobuccal and palatal root canals (8.3%).

**Fig. 1:**
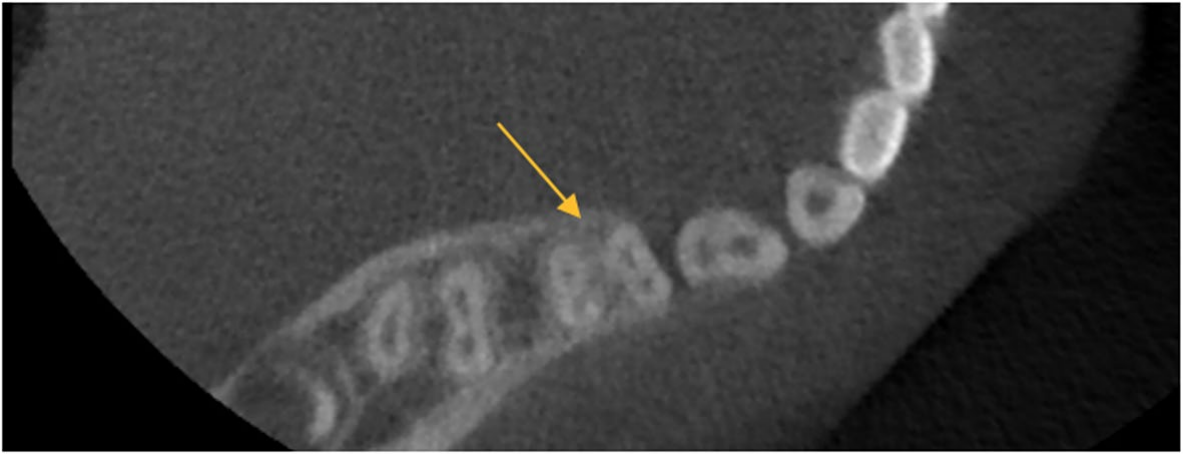
Axial view of primary second molar shows the two-rooted tooth with two canals in each

**Fig. 2:**
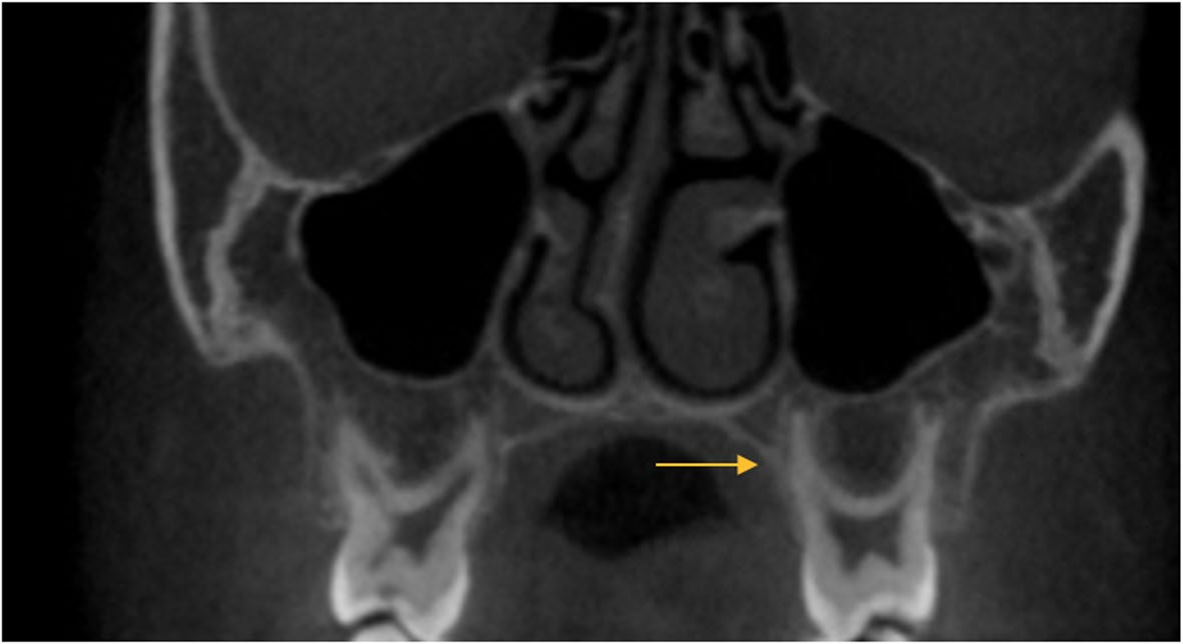
Coronal view of primary molars shows buccal concavity in palatal root

Table 4 shows the frequency distribution of root surface concavities in primary molars. A total of 23.3% of primary teeth had no surface concavity; concavity in the buccal surface was the most common (48.4%) followed by the mesial surface (23.4%). Figures 1 and 2 respectively show axial and coronal views of primary molars.

**Table 4:** Frequency distribution of root surface concavities in primary molars

Surface with concavity	Frequency	Percentage
No concavity	14	23.3
Buccal	16	26.7
Lingual/palatal	8	13.3
Mesial	5	8.3
Distal	3	5
Buccal, lingual/palatal	4	6.7
Buccal and mesial	7	11.7
Buccal and distal	1	1.7
Mesial and distal	1	1.7
Buccal, mesial, and distal	1	1.7
Total	60	100

Independent t-test was used to compare the variables between the first and second primary molars (Table 5), which showed a significant difference in the number of roots (*P* = 0.013), number of canals (*P* = 0.022), and type of mesiolingual canal (*P* = 0.034) between the primary first and second molars.

**Table 5:** Comparison of the variables between the first and second primary molars using independent t-test

Variable	Std. deviation	Degree of freedom	T statistic	*P* value
**Number of roots**	0.142	58	2.563	0.13
**Mesiobuccal canal type**	0.256	58	1.570	0.122
**Mesiolingual canal type**	0.123	58	2.171	0.034
**Mesial canal type**	0.346	58	1.587-	0.118
**Distal canal type**	0.427	58	0.489-	0.627
**Distobuccal canal type**	0.277	58	0.977	0.333
**Distolingual canal type**	0.200	58	0.622	0.536
**Palatal canal type**	0.336	58	0.190	0.850
**Number of canals**	0.142	58	2.357	0.022
**Concavity**	4.872	58	0.090	0.928
**Gender**	0.134	58	0.326-	0.745

## Discussion

This study assessed the root canal morphology of primary molars using CBCT. The results showed that the majority of maxillary right second molars (80%), mandibular left second molars (100%), mandibular left first molars (66.7%), mandibular right first molars (100%), and mandibular right second molars had four canals. Also, 50% of maxillary left second molars and maxillary right second molars had 5 canals. Moreover, the majority (60%) of maxillary left first molars had three canals. A review study by Mahesh and Nivedhitha [16] regarding the morphology of primary mandibular second molars showed that the most common morphology was presence of three canals (2 mesial and 1 distal). The present study evaluated all maxillary and mandibular molars, and in contrast to the study by Mahesh and Nivedhitha [16] over 90% of the mandibular right and left second molars in the present study had four canals.

Ozcan et al. [9] evaluated the morphology of primary root canals by CBCT in Turkey. They assessed 343 maxillary and mandibular first and second molars and revealed significant differences regarding the number of root canals among the four groups. The number of primary molar root canals ranged from 2 to 4. Maxillary molars mainly had one single canal [9]. Unlike their study, the present results showed that the majority of maxillary and mandibular first and second molars had 3 or 4 canals, and no single-canal molar tooth was found. Difference between their results and the present findings may be due to different races and sample size, since the sample size in the present study was smaller than that of Ozcan et al. [9]. Yang et al. [17] evaluated 487 CBCT scans of mandibular primary second molars in China and found that 73.31% of primary mandibular second molars had four canals, 25.26% had three canals, 0.82% had two canals, and only 3 teeth had five canals. In line with their findings, the present study showed that 100% of mandibular left second molars and 92.3% of mandibular right second molars had four canals. Only 7.7% of mandibular right second molars had 5 canals, which was in agreement with the results of Yang et al. [17]. Ahmed et al., [18] in Sudan evaluated 200 mandibular primary first and second molars, and reported that the majority of them (59%) had four canals. In the present study, 80% of maxillary right first and second molars, and maxillary left first molars, 100% of maxillary left second molars, 66.7% of mandibular left second molars, 50% of mandibular left first molars, and 61.5% of mandibular right second molars had three roots. Also, 50% of mandibular left and right first molars had two roots. The four-rooted teeth included maxillary right second molar (20%), mandibular left second molar (22.2%), mandibular right first molar (25%), and mandibular right second molar (30.8%).

Ozcan et al. [9] found significant differences among the primary maxillary and mandibular first and second molars (4 groups) in terms of the number of roots and canals, and root length. The number of roots varied from 2 to 4, and maxillary molars were mostly single-canal. In line with their results, the majority of first and second molars of both jaws had three roots in the present study.

In the current study, the majority of primary teeth did not have the mesial (85%) canal. This rate was 76.7% for the distal canal, 68.3% for the mesiolingual canal, 73.3% for the distolingual canal, and 55% for the palatal canal. Type I was the most common canal type in mesiobuccal (70%) and distobuccal (68.3%) canals. Type II had the highest frequency in the palatal (18.3%) and mesiobuccal (5%) canals. Type III was the most common type in distal (18.3%) followed by mesiobuccal root canals (3.3%). Type IV had the highest frequency in mesial root canal (11.7%) followed by distobuccal and palatal root canals (8.3%). In a review study by Mahesh and Nivedhitha [16], Vertucci’s types IV and I had the highest frequency in mesial and distal roots. In the present study, of 9 cases with mesial root types, 7 were type IV and 2 were type I. Meryem et al. [19] evaluated the micro computed tomography images of 50 primary mandibular molars and reported that type IV was the most common root canal morphology in primary mandibular first molars with a frequency of 47% in the mesial root and 41.2% in the distal root. In agreement with their findings, the present results showed that type IV was the most common root canal morphology in the mesial root of primary molars. However, type III was more frequent in the distal root. Also, Demirez et al., [20] in Turkey reported that type IV was the most common root canal morphology. The most common root canal type was types IV and II in a study by Ahmed et al. [18].

Katge and Wakpanjar [17] evaluated the root canal morphology of 120 primary molars by the clearing technique, and reported that the most common types were Vertucci’s type IV in the mesiobuccal, distobuccal, and palatal roots of primary maxillary first molars, type I in the mesiobuccal, distobuccal, and palatal roots of primary maxillary second molars, type IV in the mesial and distal roots of primary mandibular first molars, and type I in the mesial root of primary mandibular second molars.

In the present study, concavity was the most common in the buccal surface (48.4%) followed by the mesial surface (23.4%) and palatal (20%) and lingual (20%) surfaces. Esfahanian et al. [21] evaluated the anatomical and morphological characteristics of 222 maxillary and mandibular first molars and reported that in mandibular molars, the mean diameter of the orifice and the mean root length in the buccal surface were greater than the lingual surface. Also, the mean concavity of the mesial root was greater than that of distal root. Similarly, the present study showed higher frequency of concavity of the buccal root compared with lingual root. Moreover, the frequency of root concavity was greater on the mesial than distal root surface.

Precise assessment of CBCT images by two observers was a strength of this study. Future studies are required to assess the anatomical variations of other primary teeth.

## Conclusions

A wide variation exists in the number of roots and canals of maxillary and mandibular primary molars, which calls for further attention in treatment of such teeth. The majority of primary molars had four canals, over half of the maxillary left second molars had five canals, and most maxillary left first molars had three canals. The majority of maxillary and mandibular first and second molars had three roots. Half of the mandibular first molars had two roots, and over one-fifth of mandibular first and second molars and maxillary second molars had four roots. Also, concavity was noted on over two-thirds of the buccal and mesial root surfaces of maxillary and mandibular molars.

## Data Availability

The complete documentation of participant enrolled in this study belongs to the corresponding author, Elham Khoshbin, and is available only upon reasonable request.

## Abbreviation

CBCT: Cone-beam computed tomography
